# Supplementary material for: Plant growth-promoting activity of beta-propeller protein YxaL secreted from Bacillus velezensis strain GH1-13
Source: PLoS One. 2019 Apr 25;14(4):e0207968. doi: 10.1371/journal.pone.0207968 (PMC6483160; doi:10.1371/journal.pone.0207968)
Supplement: S1 Fig — The evolutionary history was inferred using the minimum evolution method in MEGA 7. The tree out of 46 minimum evolution trees (sum of branch length = 5.46420670) is shown, with branch lengths in the same units as those of the evolutionary distances used to infer the phylogenetic tree. (DOCX) [file pone.0207968.s001.docx]

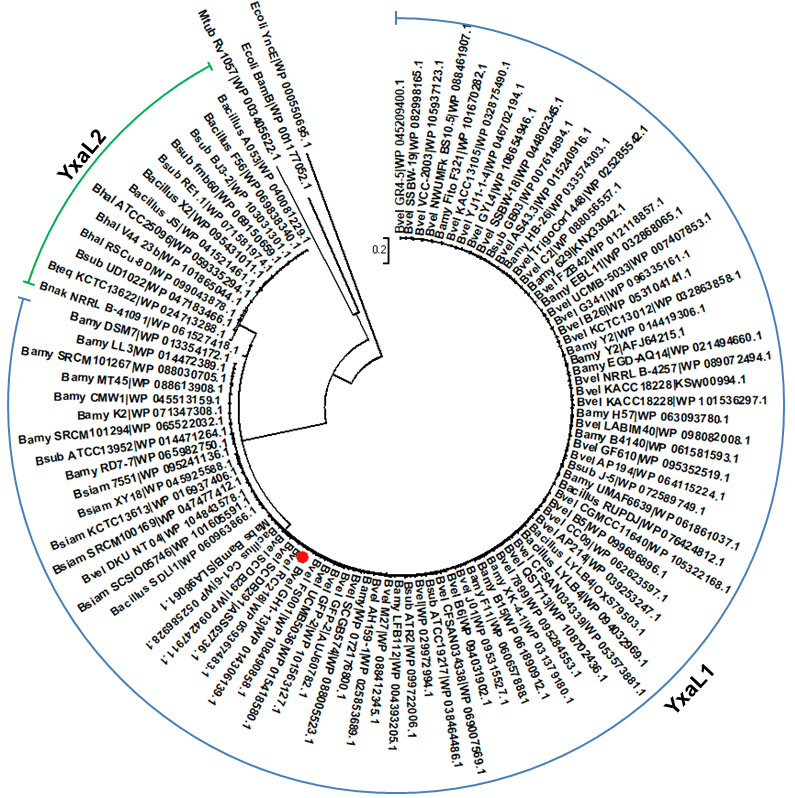


**S1 Fig. Evolutionary relationship of YxaL homologs.** The evolutionary history was inferred using the minimum evolution method in MEGA 7. The tree out of 46 minimum evolution trees (sum of branch length = 5.46420670) is shown, with branch lengths in the same units as those of the evolutionary distances used to infer the phylogenetic tree. The evolutionary distances were computed using the Dayhoff matrix-based method and are in the units of the number of amino acid substitutions per site, with the abbreviated taxonomic names shown in **Fig 1** in the main text.
