# Supplementary material for: Plant growth-promoting activity of beta-propeller protein YxaL secreted from Bacillus velezensis strain GH1-13
Source: PLoS One. 2019 Apr 25;14(4):e0207968. doi: 10.1371/journal.pone.0207968 (PMC6483160; doi:10.1371/journal.pone.0207968)
Supplement: S2 Fig — The recombinant protein YxaL with the N-His TEV cleavage site was purified by stepwise elution with high concentrations of imidazole (50 to 250 mM) and, after the N-His TEV cleavage site was removed from the recombinant protein YxaL by overnight digestion with a 1:100 ratio of a recombinant TEV protease (N-His), the mature protein YxaL was recovered in unbound fraction eluted by low concentrations of imidazole (20 to 25 mM) and the protein size and purity was determined by size exclusion chromatography and SDS PAGE. (DOCX) [file pone.0207968.s002.docx]

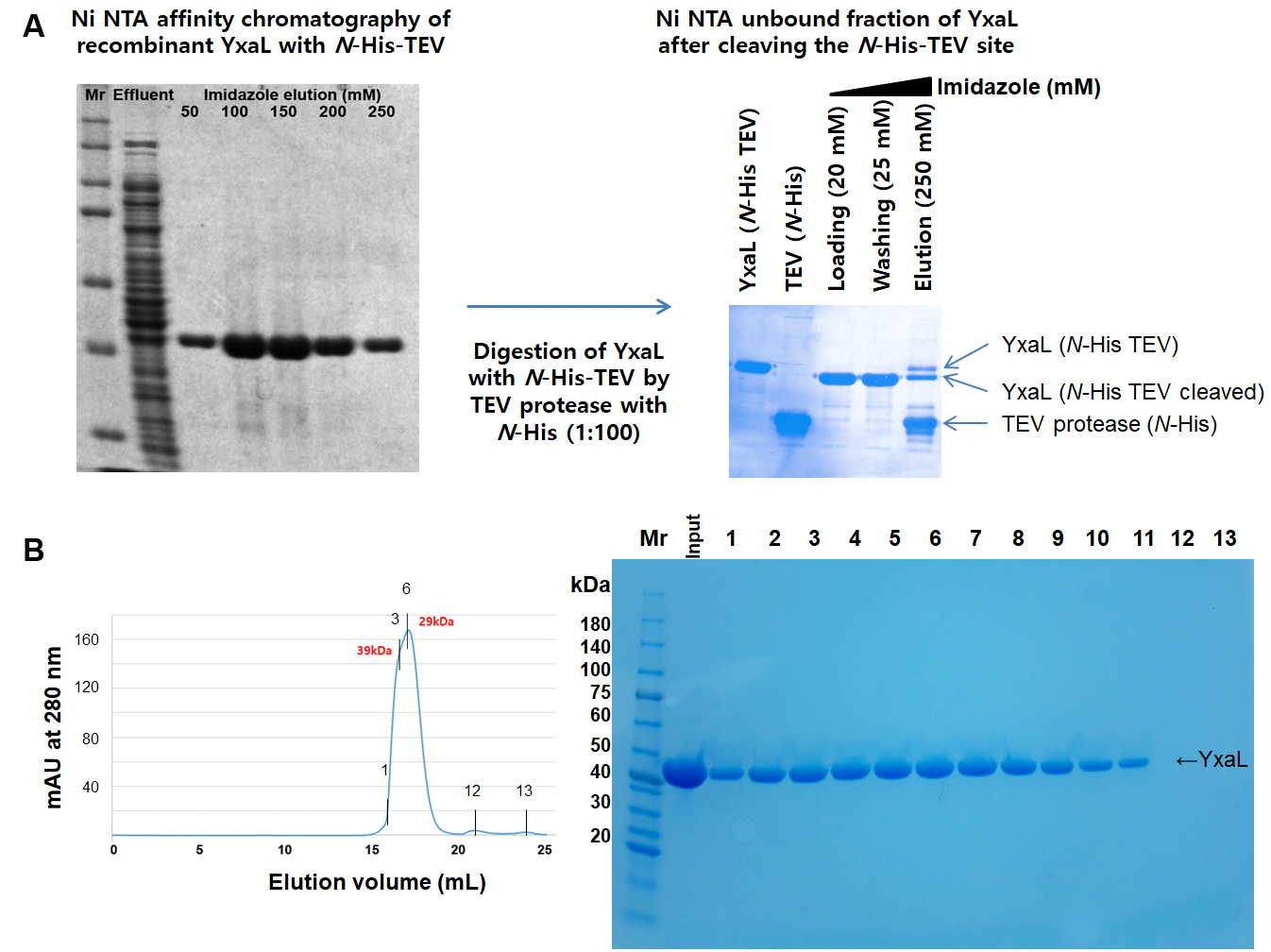


**S2 Fig. Recombinant protein production and purification of the mature YxaL protein using an Ni NTA agarose column.** A: Purification of the recombinant YxaL protein with the *N*-His TEV cleavage site by stepwise elution with high concentrations of imidazole (50 to 250 mM). After removal of the *N*-His TEV cleavage site from the recombinant protein YxaL by overnight digestion with a 1:100 ratio of a recombinant TEV protease (*N*-His) at 37 °C, the majority of purified YxaL with the original sequence was recovered in unbound fraction eluted by low concentrations of imidazole (20 to 25 mM). B: Determination of protein size and purity of purified YxaL by size exclusion chromatography and SDS PAGE.
