# Supplementary material for: Plant growth-promoting activity of beta-propeller protein YxaL secreted from Bacillus velezensis strain GH1-13
Source: PLoS One. 2019 Apr 25;14(4):e0207968. doi: 10.1371/journal.pone.0207968 (PMC6483160; doi:10.1371/journal.pone.0207968)
Supplement: S3 Fig — After planting of rice seeds soaked with various concentrations of YxaL (0 to 100 mg L-1), the primary and hair root lengths were measured on 1 week after seedling emergence. (DOCX) [file pone.0207968.s003.docx]

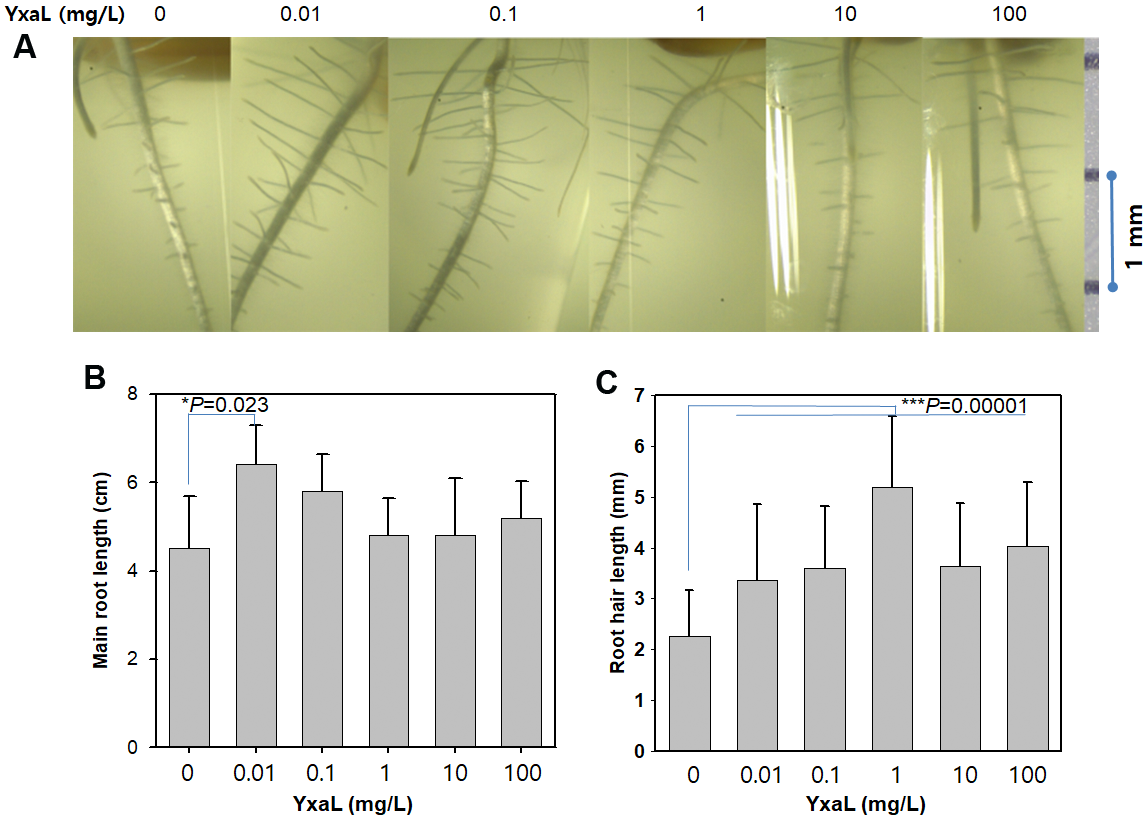


**S3 Fig. Effects of soaking seeds with YxaL on the root growth and development of rice (*Oryza sativa* L.).** A: Typical microscopic images of hair roots in 1-week-old seedlings of treated and untreated seeds (*n* = 10 for each group), which were cultured in 0.5% agarose Murashige and Skoog (MS) medium using the same method as described of the *Arabidopsis* seeds, after having been treated with purified YxaL (0 to 100 mg L^-1^) in a soaking solution, as described in the Materials and methods. B: Differences in the primary root lengths of seedlings developed after treatment of seeds with 0.01 mg L^-1^ YxaL solution compared to the untreated seeds. A significant difference between the primary root lengths of treated and untreated seedlings was determined by two-tailed *t*-test with the *P*-value of less than 0.05. C: Differences in the hair root growth in 1-week-old seedlings of treated and untreated rice seeds. Compared to the untreated group, all of the treated groups exhibited a significant improvement in hair root growth. From these results, the optimal concentration of YxaL for soaking seeds was determined to be 1 mg L^-1^.
