# Supplementary material for: Plant growth-promoting activity of beta-propeller protein YxaL secreted from Bacillus velezensis strain GH1-13
Source: PLoS One. 2019 Apr 25;14(4):e0207968. doi: 10.1371/journal.pone.0207968 (PMC6483160; doi:10.1371/journal.pone.0207968)
Supplement: S1 File — 97 homologous sequences of YxaL and 3 paralogous sequences of Mycobacterium tuberculosis Rv1057, Escherichia coli BamB and YncE were collected from the National Center for Biotechnology Information to analyse the phylogenetic trees (Figs 1 and S1) and to search the beta-propeller motif sequences (Table 1) in the consensus sequences of YxaL1 and YxaL2 generated by multiple sequence alignments. (DOCX) [file pone.0207968.s004.docx]

**Supporting Information:**

**Plant Growth-Promoting Activity of Beta-Propeller Protein YxaL Secreted from Bacillus *velezensis* Strain GH1-13**

Yong-Hak Kim^1*^, Yunhee Choi^1^, Yu Yeong Oh^1^, Nam-Chul Ha^2^, and Jaekyeong Song^3*^

^1^Department of Microbiology, Daegu Catholic University School of Medicine, Daegu, Republic of Korea

^2^Research Institute for Agriculture and Life Sciences, Center for Food and Bioconvergence, Seoul National University, Seoul, Republic of Korea

^3^Agricultural Microbiology Division, National Institute of Agricultural Sciences, Rural Development Administration, Wanju-gun, Jeollabuk-do, Republic of Korea

**Running title:** Effect of YxaL on plant growth

Correspondence: Y.H.K. ([ykim@cu.ac.kr](mailto:ykim@cu.ac.kr)) and J.S. ([mgjksong@korea.kr](mailto:mgjksong@korea.kr))

**S1 File. Consensus amino acid sequences of YxaL homologous proteins.** We collected 97 homologous sequences of YxaL and 3 paralogous sequences of *Mycobacterium tuberculosis* Rv1057, *Escherichia coli* BamB and YncE from the National Center for Biotechnology Information. From the phylogenetic analysis, YxaL homologs were divided into two types, YxaL1 and YxaL2, as shown in Fig 1 and S1 Fig. Consensus sequences of YxaL1 in 84 protein sequences and YxaL2 in 13 protein sequences were produced by multiple sequence alignments using Geneious version 10.2.3 (Kearse et al., 2012). The alignment was produced using the BLOSUM 62 algorithm to mitigate any query with low similarity (<62% conserved amino acid residues) in a protein homology group across species. The consensus sequences are given below.

>consensus sequence of YxaL1, 415 amino acid residues

MKKKTASLRMKTLAAGAAVAAALSVGAVSDLPGAKWLHPAAAQAAETVFKQNHAASGFLAGRYDAQAMSPTMFNWSRESRFTSTADGALKWEKNVPANPQNGAGAAVDGDGTVFIQSKDGKLTAYHPDGTVKWVTENLGTTYTLTPVLGTNGVIYLPSHDKKLYFIDKETGNILTSVPLSGAPSSDAAIGSDGTLYVSTLDNYIYAIKPTSPSTWTQKWKFKTNGVVGSAPVLASNGTLYTATYNNIFYAINSGTGQVKWSKTTSNGFKGYPVIDRDGTVYAGNQDGNLYAYTSTGAVKWTFPLNGFSSSSLAIDHNGNVYIGSGSGELFSISKTGNMNWSFYTDGPVRTAPLIDADGNVYFGSDDKNVYAVDADGNEKWRYQTDSNVISSPVLAEDGTLYVGTYTKLLAFGAKK

>consensus sequence of YxaL2, 394 amino acid residues

MKALIAGAAVAAAVSAGAVSDVPAAKVLQPAAAYAAETVFSQNNEASGFLTGRYDVQAMAPAMFNWSRESRFAGNTDGTLKWQNDIRTTPQNGAGAVIDGDGTVYLHSRDGEMKAFNPDGSVKWVTGNLGKTFTQSPVLGTNGVIYLASYDKKIYFIDKETGEILTTVPLSGGPSSETVIGSDGTLYFSTLDNYVHAIKPTSKSTWTERWKLKTNGVVSSVPVLAKNGTVYVGTYNNVFYAINSGTGQVKWSRTTSNAFKGYPVIDKDGTVYAGNQDGQLYAYTSTGSLKWTFPLNGFSSSSPAIDHNGNIYIGSGSGELFSISKNGDMNWSFYTDGPVRTAPLIDAKGTVYFGSDDMKVYAADANGNELWSYQTDSNVVSSPQLAEDGTLYIG

**Reference**

Kearse, M. et al. Geneious Basic: an integrated and extendable desktop software platform for the organization and analysis of sequence data. *Bioinformatics* **28**, 1647-1649 (2012).
