# Supplementary material for: Plant growth-promoting activity of beta-propeller protein YxaL secreted from Bacillus velezensis strain GH1-13
Source: PLoS One. 2019 Apr 25;14(4):e0207968. doi: 10.1371/journal.pone.0207968 (PMC6483160; doi:10.1371/journal.pone.0207968)
Supplement: S1 Table — See Figs 2 and 3 for the relative expression levels of the yxaL gene in Bacillus velezensis strain GH1-13 and plant hormone-responsive marker genes (IAA1, GH3.3, AFB4, and ACS11) in Arabidopsis thaliana normalized to the respective 16S and 18S rRNA levels by the ΔΔCq method. (DOCX) [file pone.0207968.s005.docx]

**S1 Table. Primers used in qPCR for determination of the expression levels of the *Bacillus yxaL* gene and *Arabidopsis* genes.**

| Strain/Gene names | Forward (5′→3′) | Reverse (5′→3′) |
| --- | --- | --- |
| *Bacillus velezensis* strain GH1-13 | | |
| *yxaL* | GTTTCTTGGCGGGACGGT | AGCACCGGAGTCAGCGTA |
| *16S rRNA^1^* | CCTACGGGAGGCAGCAGTAG | CAACAGAGCTTTACGATCCGAAA |
| *Arabidopsis Arabidopsis thaliana* | | |
| *GH3.3^2^* | TCGGATAAAACCGATGAAGC | TCAACGACTCCTCCATTTCC |
| *IAA1^2^* | GGAAGTCACCAATGGGCTTA | GAGATATGGAGCTCCGTCCA |
| *AFB4^2^* | AATCGAGGACGAAGAAGCAA | TCTGCATTTCCACCATTTCA |
| *ACS11^2^* | CCCACTTGGAACCTCTACCA | ATCAAGCCAACACGAAATCC |
| *18S rRNA^3^* | GCGTTTGA GAGGATGTGGCGGGGAAT | TAAATGCGTCCCTTCCATAAGTCGGG |

^1^ Huang, L. et al. Antibacterial activity of a modified unfilled resin containing a novel polymerizable quaternary ammonium salt MAE-HB. *Sci. Reports* **6**, 33858; 10.1038/srep33858 (2016).

^2^ Gleason, C., Foley, R.C. & Singh, K.B. Mutant analysis in *Arabidopsis* provides insight into the molecular mode of action of the auxinic herbicide dicamba. *PLoS ONE* **6**, e17245; 10.1371/journal.pone.0017245 (2011).

^3^ Kim, Y.K. et al. Ribosomal protein S6, a target of rapamycin, is involved in the regulation of rRNA genes by possible epigenetic changes in *Arabidopsis*. *J. Biol. Chem.* **289**, 3901-3912 (2014).
